# Effects of cannabinoids on the development of chick embryos *in ovo*

**DOI:** 10.1038/s41598-019-50004-7

**Published:** 2019-09-17

**Authors:** Sofia B. Gustafsson, Stig O. P. Jacobsson

**Affiliations:** Department of Pharmacology and Clinical Neuroscience, Umeå University, SE-901 87 Umeå, Sweden

**Keywords:** Receptor pharmacology, Toxicology

## Abstract

We have examined the effects of the synthetic cannabinoids HU 210 and HU 211, the plant-derived cannabidiol and the endogenous cannabinoid anandamide on the viability and development of chick embryos. Fertilized White Leghorn chicken eggs were injected with the test compounds or carrier vehicle, via a drilled small hole in the egg, directly into the egg yolk. After nine days of exposure, the embryonal viability, length and wet weight of embryos, and wet weight of brains were measured, and the development stages were assessed according to the Hamburger and Hamilton (HH) scale. The potent synthetic cannabinoid receptor agonist HU 210 and the non-psychotropic cannabidiol were embryotoxic at the highest concentrations examined (10 µM and 50 µM, respectively), with no viable embryos after the HU 210 injection, and 20% viability after the cannabidiol injections. The effects of HU 210 on the chick embryo were attenuated by α-tocopherol and the cannabinoid receptor antagonist AM251, whereas only α-tocopherol gave a statistically significant protection against the embryotoxic effects of cannabidiol. This study shows that exposure to plant-derived or synthetic cannabinoids during early embryonal development decreases embryonal viability. Extrapolation of data across species is of course difficult, but the data would argue against the use of cannabinoids, be it recreationally or therapeutically, during pregnancy.

## Introduction

Cannabis has a long history of medical and therapeutic use, and the discovery of the cannabinoid (CB) signalling system, comprising CB receptors, endogenous ligands and enzymes for ligand biosynthesis and inactivation, has led to an enormous expansion of the CB research field and in turn to the identification of new targets for therapeutic drugs^1–3^. Many of the wide spectrum of central and peripheral actions of CBs are mediated through CB receptors. The CB_1_ receptor was discovered in 1990^4^ and the CB_2_ receptor was cloned in 1993^5^. Both receptors are G-protein-coupled receptors, that are negatively coupled to adenylate cyclase and positively coupled to mitogen-activated protein (MAP) kinase^6^, but the CB receptors also regulate the activity of calcium and potassium channels^7^. Cannabis use is increasing in the European countries and cannabis is today the most commonly used illegal drug in Sweden. Even if the long-term health effects of cannabis use remain uncertain, epidemiological data and laboratory evidence suggest that cannabis and other CBs possess adverse effects. For example, CBs reduce reproductive function in both male and females, and may cause retarded embryo development and pregnancy failure^8^. Compounds acting at CB receptors are highly lipophilic by nature and can easily cross barriers such as the placenta barrier, and if CBs are used during pregnancy, they enter the embryo or fetus and may increase the risk of neurobehavioral changes of the young infant^9^. In this respect, the CB receptor system actively regulates cell proliferation and differentiation both *in vitro* and *in vivo*^10^, and it has been shown that feeding female rats with CB receptor ligands during pregnancy and/or lactation modify the maturation of various neurotransmitter systems during embryonic development^11,12^. Prenatal exposure to WIN 55,212-2, at doses that caused neither malformation nor overt signs of toxicity, produced a deficit in cortical glutamatergic neurotransmission, reduction in *N*-methyl-D-aspartic acid receptor activity and alterations in neuronal development in rats^13,14^. This suggests that abnormal stimulation of the CB receptors during development may cause permanent alterations in neurotransmission and thereby produce cognitive deficits. The evidence of CB-induced teratogenicity is scarce, but some studies suggest that maternal cannabis smoking is associated with low neonatal birth weight^15^.

However, the adverse effects of cannabis and cannabinoids during pregnancy need to be further evaluated since (1) the concentrations of psychoactive compounds such as Δ^9^-tetrahydrocannabinol (THC) in e.g. marijuana and hashish preparations, is continuously increasing due to breeding selection^16–18^, and (2) the use of “Spice” products, herbal mixtures added with an increasing number of different synthetic CBs that are smoked to obtain cannabis-like effects^19^, of which many lack toxicological data^20^.

Data concerning the effects of cannabinoids on the developing chick embryos are rare and represented by some studies on the role of CB_1_ receptor signaling during neurodevelopment^21^, and the effects of THC and analogues on nucleic acid synthesis^22^, neural development^23^, and the function of embryonic heart cells^24^.

In the present study, we have investigated the effects of the synthetic cannabinoids HU 210 and HU 211, the plant-derived cannabidiol (CBD) and the endogenous cannabinoid anandamide (AEA) on the viability and development of chick embryos.

The avian CB_1_ receptors have a very close homology to human CB_1_ receptors and the chicken CB_1_ receptor pharmacology, assessed in binding experiments, resembles very closely its rodent equivalent^25^. We have previously shown that the CB_1_ receptor recognition sites in the chicken brain have similar pharmacological properties to those in the rat brain^26^. The CB_1_ receptors retain functional coupling to their G-proteins, since the synthetic cannabinoid agonist CP 55,940, at nanomolar concentrations produced a robust decrease in the cAMP response to forskolin in a manner that was blocked by the selective CB_1_ receptor antagonist AM251^27^.

HU 210 is one of the most potent synthetic cannabinoids yet discovered, being 100–800 times more potent than Δ^9^-THC^28,29^, with a long duration of action (1–2 days in rhesus monkeys^30^).

HU 210 is an analogue of the tricyclic benzopyran Δ^9^-THC with reported *K*_i_ values of 0.06–1.6 nM at the CB_1_ receptor and 0.52 nM at the CB_2_ receptor^31–33^. We have previously showed that synthetic CB receptor agonists found in Spice branded products^34,35^, such as HU 210 and CP 55,940, are cytotoxic in various *in vitro* cell models^36–38^. It is therefore possible that, beside the well-known side effects of cannabis or cannabis-like drugs, prolonged and/or excessive recreational use of HU 210 may be cytotoxic to a wide range of cells in the body, especially cells undergoing rapid mitotic division such as immune cells and cells in the developing embryo. HU 211 is a nonpsychotropic enantiomer of HU 210 lacking activity at CB receptors but acts as a glutamate NMDA receptor antagonist^39^. CBD is a natural constituent of *Cannabis sativa* that lacks psychotomimetic properties. CBD is a weak, noncompetitive, and negative allosteric modulator of CB_1_ receptors^40–42^, however, even if the pharmacological action of CBD seems to not involve cannabinoid receptors, CBD exerts several important effects on the central nervous system, including anxiolytic, antipsychotic^43^ analgesic, or antiepileptic effects^44^^,^^45^. AEA, a *N*-acylethanolamine fatty acid amide, was the first endogenous ligand (endocannabinoid) to the CB receptors to be discovered^46^. The affinity of AEA to the human CB receptors have been reported to be (*K*_i_ values) 239 nM at the CB_1_ receptor and 440 nM at the CB_2_ receptor^47^. The pharmacological properties of AEA, that is rapidly degraded by the intracellular enzyme fatty acid amide hydrolase (FAAH)^48^, is characterized by actions at a number of non-cannabinoid receptor targets such as the transient receptor potential vanilloid 1 (TRPV1)^7^ and the peroxisome proliferation-activated receptor^49^.

The effects of the specific CB_1_ receptor antagonist AM251 and the antioxidant α-tocopherol were also investigated since these compounds have blocked or attenuated the antiproliferative/cytotoxic effects of CBs in various *in vitro* cell models^36–38,50^.

We can show that the potent synthetic CB receptor agonist HU 210 as well as the plant-derived CBD, with low CB receptor activity, are embryotoxic in a manner that could be attenuated by 100 µM α**-**tocopherol or by 1 µM AM251, when injected *in ovo*.

## Results

To examine the concentration-dependent effects of CBs on the development and survival of chick embryos, the test compounds were injected directly into the egg yolk. Of 305 eggs used in the study, 40 eggs were injected with saline, 41 eggs with DMSO, 12 eggs with ethanol, and 212 eggs with the test compounds at day 1, 4 and 7 after incubation. At incubation day 10 (corresponds approximately to stage 36 according to the HH scale), 290 eggs were examined for weight and vascularization of the egg, embryonal mean crown-rump (C-R) length, mean wet embryonal weight, and mean wet brain weight (Table 1). The mean weight loss of fertilized eggs injected with saline and incubated for 10 days was −2.53 ± 0.07 g (mean ± SEM). None of the injected compounds significantly affected the egg weight loss.

**Table 1:** Concentration-dependent effects of cannabinoids on the vascularization of the egg, and the mean crown-rump (C-R) length, mean wet body weight and mean wet brain weight of chick embryos incubated for 10 days.

Treatment	Vascularization (mean score)	C-R length (cm)	Wet embryo weight (g)	Wet brain weight (mg)
Saline	1.97 ± 0.03 (37)	3.17 ± 0.15 (37)	1.97 ± 0.13 (37)	126 ± 7 (37)
DMSO (0.05%)	1.95 ± 0.03 (41)	3.15 ± 0.13 (41)	1.97 ± 0.08 (41)	122 ± 5 (41)
EtOH (0.05%)	1.92 ± 0.08 (12)	3.11 ± 0.11 (12)	1.87 ± 0.03 (13)	107 ± 1 (13)
α-Toc. (100 µM)	1.78 ± 0.15 (9)	2.51 ± 0.38 (9)	1.33 ± 0.25 (9)	88 ± 17 (9)
AM251 (1 µM)	1.92 ± 0.08 (12)	2.88 ± 0.23 (12)	1.47 ± 0.15 (12)	97 ± 10 (12)
HU 210				
1 µM	1.77 ± 0.17 (13)	2.97 ± 0.27 (13)	2.02 ± 0.24 (13)	118 ± 13 (13)
3 µM	1.77 ± 0.17 (13)	3.20 ± 0.17 (13)	1.40 ± 0.25 (13)	88 ± 6 (13)
10 µM	1.16 ± 0.16 (12)^§^	1.46 ± 0.21 (26)^§^	0.42 ± 0.12 (26)^§^	32 ± 9 (26)^§^
10 µM + α-Toc.	1.78 ± 0.15 (9)	2.50 ± 0.42 (9)	1.35 ± 0.27 (9)^*^	91 ± 18 (9)^*^
10 µM + AM251	1.78 ± 0.15 (9)	2.69 ± 0.39 (9)	1.48 ± 0.28 (9)^†^	92 ± 18 (9)^*^
HU 211				
1 µM	2 (7)	3.07 ± 0.29 (7)	1.68 ± 0.32 (7)	107 ± 15 (6)
3 µM	2 (7)	2.81 ± 0.38 (7)	1.48 ± 0.38 (7)	94 ± 18 (6)
10 µM	2 (7)	2.99 ± 0.33 (7)	1.68 ± 0.29 (7)	106 ± 22 (6)
CBD				
5 µM	1.60 ± 0.22 (10)	2.07 ± 0.48 (10)	1.31 ± 0.35 (10)	74 ± 21 (10)
20 µM	1.40 ± 0.22 (10)^*^	1.83 ± 0.55 (10)^†^	1.11 ± 0.37 (10)^*^	67 ± 22 (10)^*^
50 µM	0.95 ± 0.18 (10)^§^	0.95 ± 0.32 (19)^§^	0.52 ± 0.21 (19)^§^	35 ± 14 (10)^§^
50 µM + α-Toc.	1.89 ± 0.11 (9)^‡^	2.79 ± 0.40 (9)^‡^	1.66 ± 0.25 (9)^†^	121 ± 12 (8)^‡^
50 µM + AM251	1.67 ± 0.17 (9)	2.14 ± 0.45 (9)	1.29 ± 0.32 (9)	106 ± 20 (8)^†^
AEA				
5 µM	2 (10)	3.16 ± 0.35 (10)	2.23 ± 0.06 (10)	135 ± 5 (10)
20 µM	2 (10)	3.13 ± 0.24 (10)	1.63 ± 0.25 (10)	115 ± 15 (10)
50 µM	2 (10)	3.08 ± 0.35 (10)	1.83 ± 0.22 (10)	116 ± 14 (10)

All data are means ± SEM with number of eggs examined in brackets. Statistically significant differences (analysed by one-way ANOVA with *post-hoc* Bonferroni’s multiple comparisons test for the C-R length, embryo and brain weight data, or Kruskal-Wallis nonparametric test with Dunn’s test for multiple comparisons for the vascularization scores) from corresponding vehicle treatment (0.05% DMSO or EtOH), or when the combination of CB + α-Toc. or AM251 are compared with CB treatment *per se*, are indicated as: ^*^P < 0.05, ^†^*P* < 0.01, ^‡^*P* < 0.001, or ^§^*P* < 0.0001.

Embryonal viability and the Hamilton and Hamburger development stage were assessed based on the embryonal length and weight measurements, and overall 88% viable embryos were found in the saline control group with an average HH stage of 35 ± 0.83 (means ± SEM), 83% viability when exposed to DMSO (HH stage 34 ± 1.1), and ethanol treatment resulted in 89% viability (HH stage 35 ± 1.6). Upon visual examination, no gross morphological malformations of e.g. limbs, heart, beak, brain or eyes were observed in CB-treated chick embryos or in the controls.

**Figure 1:**
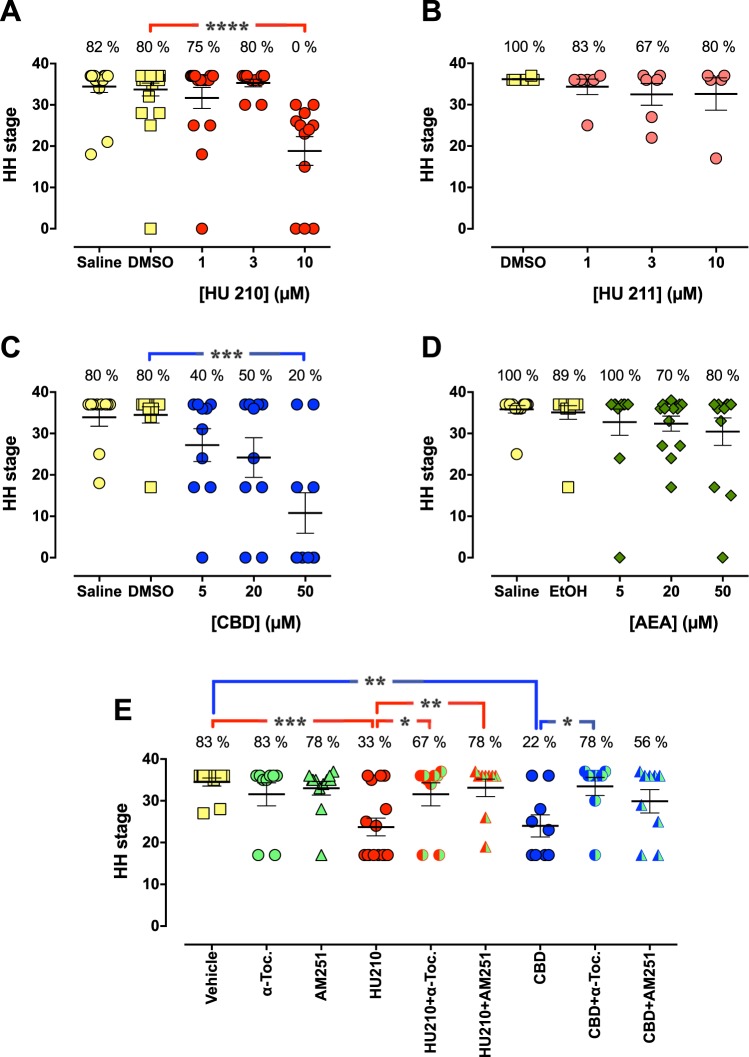
Concentration-dependent effects of HU 210 (**A**,**E**), HU 211 (**B**), CBD (**C**,**E**), and AEA (**D**) on the development and survival of chick embryos. The protective effects of 100 µM α-tocopherol (α-Toc.) or 1 µM AM251 against the embryotoxic effects produced by 10 µM HU 210 or 50 µM CBD are shown in (**E**). The embryos were treated *in ovo* with the test compounds by repeated injections (after 1, 4 and 7 days) in the yolk. On day 10, the eggs were cracked and embryos were analyzed in terms of their viability (as indicated in panels as percentage live embryos) and anatomical stage according to the Hamburger-Hamilton scale. Shown are dot plots with means ± SEM of 6–25 individual embryos per treatment. Statistically significant differences (one-way ANOVA with post-hoc Dunnett´s multiple comparison test) are indicated as: *P < 0.05, **P < 0.01, ***P < 0.001, or ****P < 0.0001, when the HH stage at each CB treatment group is compared with corresponding vehicle (0.05% DMSO or EtOH) controls, and in (**E**) when the combination of CB + α-Toc. or AM251 are compared with CB treatment *per se*.

The synthetic CB HU 210 (Fig. 1A), at a concentration of 10 µM but not at 1 µM or 3 µM, killed all chick embryos (*n* = 12) and the average HH stage that had been reached was only 19 ± 3.5 (*P* < 0.0001 vs. control DMSO treatment). This stage corresponds to approximately day 2–3 according to the HH scale^51^. Thus at least 50% of the embryos died after the first injection of 10 µM HU 210, and after the second injection (day 4) all embryos were dead. HU 211 did not produce any statistically significant effects on the chick embryos (Fig. 1B). On the other hand, CBD showed dose-dependent effects on both the viability and the development (Fig. 1C; *P* = 0.0003). The viability dropped 50% at a concentration of 5 µM and the HH stage decreased to 27 ± 4.0 (corresponding to approximately day 5 according to the HH scale), and at 50 µM, the viability was 20% and the HH stage only 11 ± 4.9 (*n* = 10; *P* < 0.001 when compared to DMSO controls). AEA showed a weak, but statistically insignificant, dose-dependent effect. At 50 µM, the viability was 80% and the HH stage 30 ± 3.3 (Fig. 1D).

In a subsequent series of experiments, we tested the effects of the CB_1_ receptor antagonist AM251 and the antioxidant α-tocopherol on the embryotoxicity produced by HU 210 and CBD. Neither α-tocopherol (100 µM), nor AM 251 (1 µM) had any statistically significant effects *per se* on the viability and development of chick embryos (Table 1 and Fig. 1E). However, both compounds protected against the embryotoxicity produced by 10 µM HU 210, since the embryo viability increased from 33% in eggs treated with HU 210 to 67% in eggs injected with HU 210 + or α-tocopherol, and 78% when injected with the combination of HU 210 and AM251. The HH stage also increased from 24 ± 2.1 (HU 210 *per se*), to 32 ± 2.8 (*P* < 0.05) and 33 ± 2.1 (*P* < 0.01), respectively. α-Tocopherol also protected against the toxicity produced by 50 µM CBD (22% viability; HH stage 24 ± 2.6; *P* < 0.01 vs. DMSO controls) by increasing the viability to 78% and restoring the HH stage to 33 ± 2.2 (*P* < 0.05 vs. CBD *per se*). AM 251 did not produce a statistically significant protection against the embryotoxic effect of CBD. The vascularization score of the chicken eggs followed the pattern of the HH stages, with an average of 1.97 ± 0.03 in eggs injected with saline that were significantly reduced to 1.16 ± 0.16 in eggs injected with 10 µM HU 210 and 0.95 ± 0.18 in eggs injected with 50 µM CBD (Table 1).

## Discussion

Cannabis, such as marijuana, is the most widely used illicit drug by pregnant women. Exposure to CBs, be it recreationally or therapeutically, may cause adverse effects on reproductive function and pregnancy. It has been shown that the major psychoactive ingredient in cannabis, Δ^9^-THC, not only diffuse across the placenta but also accumulates in the fetus^52^. The outcome of prenatal cannabis use in humans is however uncertain and the few studies performed are largely contradictory^53^. Early case reports have not been supported by large well-controlled epidemiological studies, but there is suggestive evidence that infants exposed *in utero* to cannabis have behavioural and developmental effects during the first few months after birth and that these continue in subtle forms up to adulthood^9,54^. Studies have shown that maternal cannabis use during pregnancy^55,56^ is associated with growth restriction in mid and late pregnancy, and lower birthweight, while no such association was found for paternal cannabis use in the same period, demonstrating a direct biological effect of maternal intrauterine exposure to cannabis on fetal growth^57^. However, in animal models (e.g. mouse, rat, and rabbit) the developmental toxicity of cannabinoids is more established, especially neurobehavioral abnormalities in the exposed offspring. A wide array of animal studies has been performed to dissect the molecular, physiological and behavioural effects of prenatal cannabis exposure. In rats, prenatal cannabinoid exposure results in a range of effects from axonal bundle malformation^58^ to lower memory function and motor hyperactivity^59^.

We have chosen to use the chick embryo model system to study the effects of CBs on embryonal development. It is an ideal model that corresponds to the first month of embryonal development in mammals and is well suited to the investigation of the effects of chemicals on the development of embryos. The possibility to window the egg, directly inject the test compounds and thereby exactly target exposures to specific developmental stages, followed by examination of the embryo, makes the chicken embryo model system advantageous for developmental biology, but also experimental toxicology and teratology.

Since drug-induced changes in maternal and placental physiology are eliminated, the model is especially useful for studies focusing on the molecular mechanisms by which drugs alter embryonal development. We have used *in ovo* yolk injections to administer the compounds. This method has been shown to be superior in order to minimize perturbations to the embryos and to decrease the occurrence of gross morphological abnormalities, induced by the treatment mode *per se*^60^.

It has been shown that the cannabinoid CB_1_ receptor expression spatially and temporally follows neuronal differentiation in the early chick embryo^61^, and we have previously shown that the pharmacological properties of the chick CB_1_ receptor are broadly similar to the rodent counterpart^26^. By using the CB_1_ receptor-selective antagonist radioligand [^3^H]SR 141716 A we found that chicken brain CB_1_ receptors are pharmacologically similar to rat brain CB_1_ receptors, and that rates of association and dissociation, K_D_ values and inhibition by a variety of cannabinoid ligands were very similar in membranes from chicken and rat brain^26^. In addition, the shift to the right of the inhibition of [^3^H]SR 141716A binding by CP 55,940 in the presence of nonhydrolysable GTP analogues^26,27^ is in line with a previous studies in the rat brain, and would suggest that in the chicken membranes, the CB_1_ receptors retain functional coupling to their G-proteins.

In present study, the synthetic CB HU 210 and the plant-derived CBD showed embryotoxic, rather than teratotoxic, effects in the chick embryo model system. Psychoyos *et al*.^23^ showed that the water-soluble analogue of Δ^9^-THC, O-2545, caused impaired formation of brain, heart, somite, and spinal cord primordial, during early embryogenesis in the chick embryo^23^. The ability of a CB_1_ receptor antagonist to block these effects was, however, not investigated. This is of importance, given that cannabinoids can affect cell viability by both CB receptor-dependent and -independent mechanisms^62^.

We could not observe any gross structural abnormalities of embryonal skeletal system such as beak deformities, absent, supernumerary or deformed limbs, nor malformations of the heart, brain or eyes in the CB-treated embryos. This is supported by a study from 1976, where THC reduced the embryonal body weight without causing any teratological effects, when injection into yolk sac of chicken eggs at various times of incubation^22^. One explanation to this discrepancy is that the CBs induce early (after the first injection at day 1) teratological damage that we have been unable to observe at day 10 due to an early embryonic death.

HU 210 produced a pronounced embryotoxicity at the added concentration of 10 µM. In contrast, the enantiomer HU 211, which lacks CB receptor-mediated effects, was without embryotoxic effects. This implicates the involvement of CB_1_ receptor in the effects of HU 210 and the conclusion is further supported by the finding that the CB_1_ receptor antagonist AM251 reduces the toxicity produced by HU 210.

The endogenous CB ligand AEA, on the other hand, was without effect upon embryonic development at the concentrations tested. AEA is, however, avidly metabolised by the enzyme fatty acid amide hydrolase, which is present in the chicken^63^, and this presumably limits its embryotoxicity. In future studies, it would be instructive to determine whether or not embryonic toxicity is seen when this enzyme is inhibited, although in general the enzyme is less sensitive to pharmacological inhibition than its rodent counterpart^63^, which may be a complicating factor.

CBD, with reported weak CB receptor activity but approved by the Food and Drug Administration for treatment of children with refractory epilepsy^64^, also induced embryotoxicity, albeit at a concentration that was five times higher than HU 210.

The teratogenic potential of CBD has been evaluated in zebrafish, and it was found that CBD did not cause any malformations of the embryos but delayed the hatching compared to control embryos^65^. In the present study, the embryotoxicity by CBD was not significantly blocked by AM251 but was sensitive to the antioxidant α-tocopherol (as was that of HU 210). CBD has been reported both to act as a protective antioxidant^66^ and as a toxic agent producing an increase in reactive oxygen species^67^ *in vitro*. Our data would suggest that the latter mechanism may be operative in the chick embryo, although as a caveat it should be pointed out that α-tocopherol is by no means a specific antioxidant since it demonstrates effects that are unrelated to the antioxidant activity and possibly reflect specific interactions with enzymes, structural proteins, lipids and transcription factors^68,69^.

The most obvious mechanistical explanation to the anti-embryotoxic effects of α-tocopherol would be its antioxidative properties. However, we have published data showing that α-tocopherol has the ability to protect against the antiproliferative or cytotoxic effects of cannabinoids in human colorectal carcinoma cancer cells^37^, rat C6 glioma cells^36,50^, and mouse embryonal P19 teratocarcinoma cells^38^, whereas other non-specific antioxidants as *N*-acetyl-L-cysteine, *N*-nitroarginine methyl ester or ascorbic acid have failed to protect the cells. α-Tocopherol has even attenuated CB-induced toxicity in C6 cells in the absence of large-scale lipid peroxidation. There are numerous mechanistical explanations for the protective effects of α-tocopherol, since this compound can interact as a ligand at certain receptors and transcription factors and has been shown to inhibit protein kinase C, 5-lipoxygenase and phospholipase A_2_ and activate protein phosphatase 2A and diacylglycerol kinase^70^. Some genes (e. g. scavenger receptors, α-TTP, α-tropomyosin, matrix metalloproteinase-19 and collagenase) are modulated by α-tocopherol at the transcriptional level^70^. α-Tocopherol also inhibits cell proliferation^71^, platelet aggregation^72^ and monocyte adhesion^73^.

However, absence of evidence is not evidence of absence, since the results do not rule out a reasonable conclusion to make based on our findings when taken together that the CBs produce their embryotoxic effects, at least in part, as a result of oxidative stress.

However, this conclusion must be validated in further experiments.

A final note concerns whether the concentrations used are pharmacologically relevant. In the study investigating the embryotoxic effects of O-2545, Psychoyos *et al*.^23^ reported that their concentration range “correspond to levels approximately 60- to 116-fold higher than levels of Δ^9^-THC detected in humans following inhalation of a single marijuana cigarette with 3.55–20% Δ^9^-THC content^74^, and thus reflects the concentrations of cannabinoid found in chronic cannabis users”. We have used much lower concentrations of CBs in our study, but it is difficult to discuss about clinically relevant concentrations since there are, to our best knowledge, no data available on concentrations found in human beings of e.g. HU 210 after consumption of Spice. It is of course important to be careful when attempting to interpret our findings in the chicken embryo to the human conditions, but since basic molecular and cellular control of early organogenesis is highly evolutionary conserved among species, the results obtained suggest a risk of cannabinoid consumption during pregnancy that may cause early miscarriage or developmental disturbances. This is important when considering the current discussion about legalisation of medical marijuana and therapeutic application of cannabinoid-based drugs.

## Methods

### Chemicals

Cannabidiol (2-((1S,6S)-3-methyl-6-(prop-1-en-2-yl)cyclohex-2-enyl)-5-pentylbenzene-1,3-diol), and HU 210 ((6aR)-trans-3-(1,1-dimethylheptyl)-6a,7,10,0a-tetrahydro-1-hydroxy-6,6-dimethyl-6H-dibenzo[b.d]pyran-9-methanol) were obtained from Tocris Cookson (Bristol, UK). Anandamide (*N-*(2-hydroxyethyl)-5Z,8Z,11Z,14Z-eicosatetraenamide), and HU 211 (3-(1,1-dimethylheptyl)-6aS,7,10,10aS- tetrahydro-1-hydroxy-6,6-dimethyl-6H- dibenzo[b,d]pyran-9-methanol; dexanabinol) were purchased from Cayman Chemical Co. (Ann Arbor, MI, USA). DMSO (dimethyl sulfoxide) were obtained from Sigma-Aldrich Sweden AB (Stockholm, Sweden). Alcohol prep pads (70% isopropyl alcohol) and saline were from Apoteket AB (Umeå, Sweden).

### Handling of chick embryos and drug administration

Fertilized White Leghorn chicken eggs (Gallus gallus), obtained from Agrisera AB (Vännäs, Sweden), were weighted and incubated with the blunt end up in an humified egg incubator (Agroswede, Malmö, Sweden) at 38 °C. The eggs were turned automatically every two hours. Before incubation, the eggs were weighed, cleaned with 70% ethanol to decrease external contamination and marked with a graphite pencil. After 24 hours of incubation, a six mm hole was made in the eggs using a Co/Tech mini grinder drill (Clas Ohlson, Sweden), and test compounds were injected with a sterile 27G 1½ needle into the yolk, carefully without harming the embryo. The hole was sealed with PVC-tape (Clas Ohlson, Sweden) between the injections. The injections were repeated day 4 and 7 and before each injection the eggs were cleaned with 70% ethanol. CBs are highly lipophilic and thus requires solubilization with DMSO, ethanol, acetonitril or surfactant agents, all of which are potentially embryotoxic. To overcome this problem, we have kept the vehicle concentration to approximately 0.05% (v/v) by serial dilution of the cannabinoids in anhydrous DMSO (≥99.9%) and α-tocopherol in ethanol (≥95%). Each stock solution was then diluted in saline to final concentrations of which 100 µl was injected into the yolk. All statistical comparisons are made against vehicle controls, i.e. eggs injected with 100 µl of 0.0498% (v/v) DMSO or 0.0475% (v/v) EtOH. Fertilized chick eggs continuously loose about 0.6–0.7% in weight per day due to evaporation of water from the eggs^75^. This is essential for the formation of the air cell and to facilitate optimised water and mineral balances in the different embryonic compartments formed during embryonic development. All eggs examined were weighted before incubation and on the day of analysis (day 10). After 10 days of incubation, the eggs were cracked and the vascularization (i.e. amount of blood vessels and capillaries) of the yolk sac and the chorioallantoic membrane (CAM) was visually examined. The vascularization was scored 0–2, where 0 is no vascularization, 1 small blood vessels with fewer capillaries, and 2 normal large blood vessels with extensive capillary network evenly distributed over the CAM surface (see Supplementary Fig. S1).

The embryos were removed from the eggshell and dissected from the extra-embryonic membranes, and viability, gross morphological abnormalities, wet embryo weights, crown-rump lengths and wet brain (optic lobes and cerebral hemispheres) weights were examined. Based on these measurements, the Hamilton and Hamburger stage (which go from 1 to 46) were determined. The Hamilton and Hamburger stages describe the structural development of the chick embryo and thereby separate morphological from temporal development, since developmental status for a given chronological age can vary widely^51^. This study was reviewed by the Regional Animal Ethics Review Board in Umeå (ref. no. A 15-08), who returned it with an advisory opinion: because the chick embryos were taken before day 12, ethical approval was not required according to Swedish law (SFS 1988:539; SJVFS 2015:24).

### Statistics

Statistical differences with respect to the appropriate controls were assessed by one-way ANOVA with *post-hoc* Bonferroni’s multiple comparisons test for the C-R length, embryo and brain weight data, *post-hoc* Dunnett´s multiple comparison test for the Hamilton-Hamburger scores, and Kruskal-Wallis nonparametric test with Dunn’s test for multiple comparisons for the vascularization scores using the statistical package built into the GraphPad Prism computer programme (GraphPad Software Inc., San Diego, CA, USA).

## Supplementary information

Supplementary Figure S1
