# Supplementary material for: Effects of cannabinoids on the development of chick embryos in ovo
Source: Sci Rep. 2019 Sep 17;9:13486. doi: 10.1038/s41598-019-50004-7 (PMC6748917; doi:10.1038/s41598-019-50004-7)
Supplement: Supplementary file 1 — Supplementary Figure S1 [file 41598_2019_50004_MOESM1_ESM.pdf]

Supplementary information

Effects of cannabinoids on the development of chick embryos *in ovo*

Sofia B. Gustafsson[1], and Stig O. P. Jacobsson[1,*]

[1]Department of Pharmacology and Clinical Neuroscience, Umeå University, SE-901 87 Umeå, Sweden

[*]stig.jacobsson@umu.se

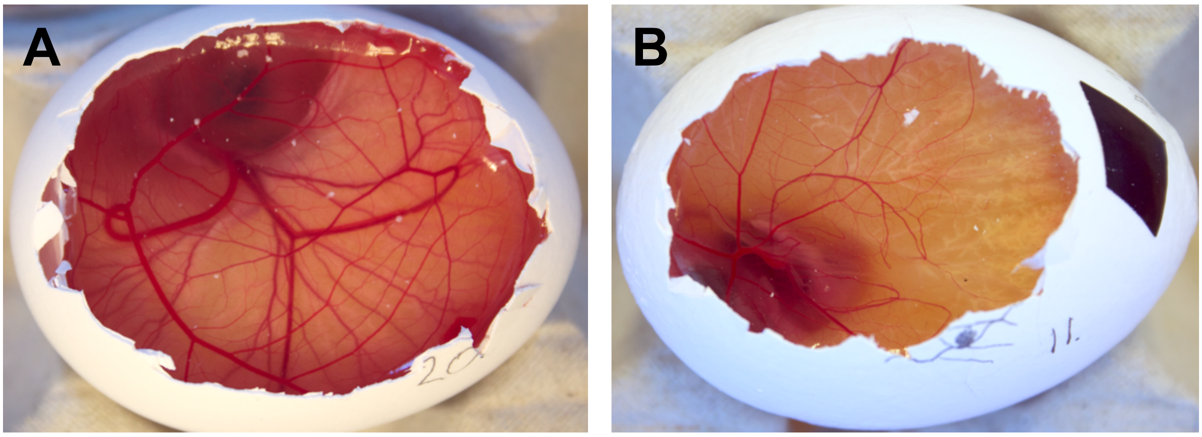

Supplementary Figure S1. Representative photographs of the vascularization in fertilized chicken eggs after 10 days of incubation. The eggs were repeatedly injected *in ovo* (after 1, 4 and 7 days) with (**A**) saline or (**B**) 20 μM CBD. After 10 days of incubation, the eggs were cracked and the amount of blood vessels and capillaries of the yolk sac and the chorioallantoic membrane was scored 0-2 (0 = no vascularization, 1 = small blood vessels with fewer capillaries, and 2 = normal large blood vessels with extensive capillary network evenly distributed. The vascularization in panel **A** is scored 2 and in panel **B** scored 1.
